# Administration of Acids, Alkalies, Etc.

**Published:** 1888-11

**Authors:** 


### ADMINISTRATION OF ACIDS, ALKALIES, ETC.

Alkalies should be given before food. Iodine and iodides should be given on an empty stomach, when they rapidly diffuse into the blood. If given during digestion, the acids and starch alter and weaken their action. Acids, as a rule, should be given between the digestive acts, because the mucous membrane of the stomach is in a favorable condition for the diffusion of the acid into the blood. Acids may be given before food when prescribed to check the excessive formation of the acids of the gastric juice. By giving it before meals, you check the osmosis stomachward of the acid-forming material. Irritating and dangerous drugs should be given directly after food, such as the salts of arsenic, copper, zinc, and iron, except where local conditions require their administration in small doses before food. Oxide and nitrate of silver should be given after the process of digestion has ended; if given with food, chemical reactions destroy or impair their special attributes, and defeat the object for which they were prescribed. Metallic salts, especially corrosive sublimate, also tannin and pure alcohol, impair the digestive power of the active principle of the gastric juice, so should be taken into the stomach during its period of inactivity. Malt extracts, cod liver oil, phosphates, etc., should be given with or directly after food, so that they may enter the blood with the products of digestion.

